# Are there distinct subtypes of developmental dyslexia?

**DOI:** 10.3389/fnbeh.2024.1512892

**Published:** 2025-01-03

**Authors:** Maria Chalmpe, Filippos Vlachos

**Affiliations:** ^1^Department of Special Education, University of Thessaly, Volos, Greece; ^2^School of Humanities, Hellenic Open University, Patras, Greece

**Keywords:** developmental dyslexia, subtypes, multiple deficits models, primary-aged students, neurocognitive deficits

## Abstract

**Introduction:**

The aim of this study was to identify if children with dyslexia can be distinguished into discrete categories based on their domain deficits, indicating various neurocognitive subtypes of developmental dyslexia (DD).

**Methods:**

The sample included 101 students in the 3rd, 4th, 5th, and 6th grades of primary school (mean age 11.15 years) with a diagnosis of dyslexia from a public center and Greek as their native language. The students underwent tests assessing a wide range of abilities, specifically phonological, memory, attention, processing speed abilities, motor, visual, and visual-motor skills.

**Results:**

Cluster analysis revealed that children with dyslexia can be divided into three subtypes. The first subtype includes children identified based on their performance in tasks evaluating the phonological abilities, memory, attention, processing speed, along with visual-motor and visual skills. The second subtype comprises children identified based on their performance in memory abilities, motor, and visual-motor skills. The third subtype includes children identified solely based on their performance in the motor skills domain.

**Discussion:**

In conclusion, our findings suggest that school-aged children with DD can be categorized into different subtypes with distinct neurocognitive characteristics. Furthermore, the results indicate that most children with dyslexia experience difficulties in more than one cognitive, sensory or motor domains, supporting contemporary models regarding the existence of multiple neurocognitive deficits in DD.

## 1 Introduction

Developmental dyslexia (DD) is a mild neurodevelopmental disorder (Brimo et al., 2021; Centanni, 2020; Ramus, 2004) with neurobiological (Lindgren et al., 1985; Kim, 2021; Vlachos, 2010) and genetic basis (Gialluisi et al., 2021; Schulte-Körne et al., 2001). It manifests as a specific learning difficulty in written language (Beidas et al., 2013), primarily in reading, and is not related to the absence of adequate education, non-typical intelligence, sensory deficits, or a potentially adverse socio-economic environment (Horowitz-Kraus et al., 2014; Nopola-Hemmi et al., 2001; Peterson and Pennington, 2015; Zoubrinetzky et al., 2014). In Greece, its prevalence rate is particularly significant, reaching approximately 5.5% (Vlachos et al., 2013).

Over approximately 50 years of systematic study on this specific disorder, various theoretical approaches have been formulated regarding its causes. Most of these approaches focus on single-deficit and can be distinguished into two levels: the biological and the cognitive.

The supporters of cognitive approaches attempt to identify the underlying deficit that causes difficulties in individuals with dyslexia and interpret them through differences observed in cognitive functions. According to the phonological deficit hypothesis (Stanovich, 1988; Vellutino and Fletcher, 2005), DD is considered the result of a deficit in phonological processing and involves three main domains: phonological awareness, verbal short-term memory, and naming speed. Difficulties in phonological processing appear to lead to problems in written language, particularly in reading. Supporters of this hypothesis argue that phonological processing deficits constitute the core impairment for most individuals with dyslexia, serving as the primary cause of the difficulties they face in both reading and writing (Snowling, 1995, 2000). However, more recent studies report the existence of other deficits, such as visual, auditory, and motor deficits in dyslexics (Heim et al., 2008; Heim and Grande, 2012; White et al., 2006) and argue that the parallel existence of these difficulties with the phonological deficit cannot be fully explained by the hypothesis of a unique deficit, the phonological, as the cause of dyslexia.

According to the hypothesis of temporal processing deficit, individuals with dyslexia seem to struggle with adequate processing of visual and/or auditory stimuli, especially when these stimuli alternate rapidly (Farmer and Klein, 1995; Tallal et al., 1985). Specifically, the various levels of difficulties faced by children with dyslexia could be attributed to a central and fundamental deficit, which is related to the brain’s ability to process the rhythm and temporal characteristics of stimuli (Vlachos, 2010). Consequently, the phonological, visual, and motor difficulties observed in children with dyslexia may be attributed to a more general difficulty in the temporal processing of stimuli (Meilleur et al., 2020; Vlachos, 2010).

Furthermore, according to the double deficit hypothesis, individuals with dyslexia exhibit deficits both in phonology and the speed of word or object naming (Wolf and Bowers, 1999). Advocates of this hypothesis argue that rapid naming is not part of phonological processing (Wolf et al., 2000) and serves as a significant predictive factor for DD (Koponen et al., 2013; Pennington, 2002). It is suggested that individuals with deficits in both domains are more likely to face severe reading problems compared to those with a single deficit (Badian, 1997; Nicolson and Fawcett, 2019). Longitudinal studies by Papadopoulos et al. (2009), as well as research by Constantinidou and Stainthorp (2009) have supported the double deficit hypothesis, demonstrating a connection between phonological deficits and processing speed. Additionally, individuals with dyslexia sometimes seem to exhibit difficulties in a wide range of skills, such as balance, motor skills, phonemic skills, and rapid sensory processing (Vlachos, 2010). These difficulties are consistent with the automaticity deficit hypothesis (Nicolson and Fawcett, 1990) according to which children with dyslexia experience fluency problems for any skill that can be made automatic through extensive practice (Vlachos, 2010). This results in individuals with dyslexia facing significant challenges in skills like reading, requiring more time and practice until they can achieve automaticity (Nicolson and Fawcett, 1990).

Moreover, many studies have linked DD to attention deficits (Bosse et al., 2007; Facoetti and Molteni, 2001). According to this hypothesis, attention deficits impact the letter encoding process, leading individuals with dyslexia to confusion in letters and visual word forms (Valdois et al., 2003). Additionally, a lack of visual attention may reduce perceptual ability (Valdois et al., 2003). The deficit in visual attention often appears to coexist with a deficit in auditory attention, which may contribute to the explanation of DD, as it can lead to difficulties in the development of phonological skills necessary for acquiring reading ability (Facoetti et al., 2003; Sperling et al., 2005). Vidyasagar and Pammer (2010) highlighted the importance of visual-spatial attention in reading. They consider that the attentional mechanisms controlled by the visual component play a significant role in letter scanning. Difficulties and deficits in this domain may cause additional problems, such as issues in the visual processing of graphemes, in the connection of graphemes to phonemes, and more generally in phonological awareness. Phonological deficits appear to stem from a deficit in visual-spatial attention. Overall, research supports the existence of deficits in various attention domains (visual, auditory, and visual-spatial) could be a factor influencing reading skills and being associated with DD.

Working memory is a high-level skill linked to a range of cognitive activities, from the simplest linguistic tasks to verbal comprehension tasks (Cowan and Alloway, 2008). It is used for the storage and processing of new information and appears to play an important role in dyslexia (McLoughlin et al., 2002). Research conducted on working memory in typically developing children has shown high performance in reading skill tasks, which was independent of performance in phonological skill tasks (Swanson, 2006). In contrast, studies examining children with dyslexia have presented findings supporting the presence of a working memory deficit, considering it one of the key characteristics defining the DD (McLoughlin et al., 2002). Children with dyslexia exhibit deficits in working memory, considering it a significant characteristic of DD (McLoughlin et al., 2002). Recent studies (Chalmpe et al., 2017; Gray et al., 2019) have also confirmed the existence of deficits in working memory in children with dyslexia.

In addition, biological hypotheses for DD were based on research, which found that dyslexia may be caused by variations in certain genes (for a review see Vlachos and Nisiotou-Mantelou, 2013), to anatomical or functional variations in certain brain regions (Ramus et al., 2018), or to variations in the ratio of gray and white matter in these regions (Vanderauwera et al., 2017). More specifically, the hypotheses of atypical structure and function in linguistic centers of the brain suggest that morphological and functional differences and abnormalities in cell architecture, mainly in areas related to language function and in the temporal fossa of the left hemisphere (around the Sylvian fissure), is related to the occurrence of DD (Cao et al., 2006). The results of functional imaging studies support this view that the most common form of dyslexia is associated with an atypically structured brain mechanism for reading (Papanicolaou et al., 2003).

Moreover, according to the magnocellular hypothesis, difficulties experienced by individuals with dyslexia in reading arise from the diminished development of magnocellular cells, which are responsible for temporal perception and motor processes (Stein, 2001; Stein and Walsh, 1997). Studies have demonstrated that individuals with dyslexia exhibit a more general magnocellular dysfunction, leading to challenges in processing sensory information, consequently hindering learning and language processing (Stein and Walsh, 1997).

Apart from the aforementioned hypotheses, the cerebellar dysfunction hypothesis suggests that the difficulties faced by dyslexic people could be a result of deficient cerebellar function (Nicolson et al., 2001). As information from the language areas of the brain and the magnocellular area in processing passes through the cerebellum, its impaired function can affect reading ability and explain the different types and degrees of dyslexia. According to Nicolson et al. (2001), a cerebellar deficit provides a reasonably satisfactory explanation for a range of problems experienced by children with dyslexia. This hypothesis predicts that a cerebellar abnormality at birth leads to mild motor and articulation problems. The lack of fluency in articulation in turn leads to a poor representation of phonological features of speech, which results in the development of difficulties in phonological awareness at around 5 years of age, leading to later problems in learning to read.

Frith (1999), considering the several hypotheses that have been put forward about dyslexia at the biological and cognitive levels, argues that there are three general causal frameworks for explaining dyslexia, which are expressed at all three levels (biological, cognitive, and behavioral). The framework of phonological deficit and dysfunction of language areas around Sylvius’ fissure suggests that dyslexia is the consequence of difficulties of linguistic origin. The magnocellular deficit framework links the sensory processing deficits exhibited by dyslexics to dysfunction of the magnocellular system. Finally, the cerebellar deficit framework links the difficulties experienced by dyslexics in developing motor and automaticity skills to cerebellar dysfunction.

However, in the last decades the development of genetics and neuroscience and the advances made by various scientific fields in understanding various developmental disorders, such as dyslexia, have challenged the single-deficit hypotheses. In more detail, no single cognitive deficit has been found that can explain all behavioral evidence of all cases of dyslexia (van Bergen et al., 2014a). In fact, research has shown that not all individuals with dyslexia have difficulties in phonological processing, and those who have problems in processing speech sounds do not necessarily have dyslexia (Peterson and Pennington, 2012; Snowling, 2008). Furthermore, as Morton and Frith (1995) pointed out, the single cognitive deficit model does not consider the situation where a biological cause, a gene, can cause a variety of cognitive deficits, which in genetics is called “pleiotropy.” In addition, the single deficit model cannot easily explain the pervasive comorbidity between different disorders, which are not independent of each other but coexist very often. According to research, DD very often coexists with Dyscalculia, attention deficit hyperactivity disorder (ADHD), specific language disorder (SLD), articulation disorder, etc. (Nisiotou and Vlachos, 2014; van Bergen et al., 2014a). Such findings have highlighted the need to move from single deficit models to approaches that argue that dyslexia may be the result of multiple cognitive deficits, as a single deficit cannot explain the great heterogeneity that characterizes this disorder, both at the etiological and behavioral level (Pavlidou et al., 2017; Pennington, 2006; Peterson and Pennington, 2015).

Pennington et al. (2012) proposed the multiple deficit model which incorporated all previous assumptions regarding the causes of dyslexia and allows the cognitive profiles of individuals with dyslexia to exhibit either a single deficit or a combination of deficits. Findings from the studies of Borleffs et al. (2018) and Pacheco et al. (2014), supported this specific model as an interpretive framework for DD.

In addition to these advances, Griffiths and Snowling (2002) argue that categorizing DD into subtypes based on reading profiles and the types of errors made by children offers a limited understanding of the characteristics of individuals with dyslexia and fails to provide meaningful insights for informing educational interventions. Contemporary perspectives argue that it would be preferable to focus on categorizing DD in terms of its neurocognitive subtypes (Zoubrinetzky et al., 2014). Studying the spectrum of neurocognitive characteristics in children with dyslexia, rather than examining only one or two hypotheses on its causes, could provide a clearer picture of the overall difficulties these children face. Consequently, it could contribute to a better diagnosis of each dyslexic child’s difficulties and play a crucial role in developing personalized intervention programs (Griffiths and Snowling, 2002).

Numerous studies have examined distinct cognitive abilities and/or sensory-motor skills related to dyslexia, but there are very few studies simultaneously exploring a broad range of cognitive and sensory-motor areas. In literature, three studies in children (Heim et al., 2008; Menghini et al., 2010; White et al., 2006) and two in adults (Ramus, 2003; Reid et al., 2007) were identified.

Specifically, Heim et al. (2008) investigated cognitive subtypes of DD in a sample of 45 students with dyslexia and 48 typically developing children from elementary schools in Germany. The study included assessments of phonological awareness, auditory discrimination, motion detection, visual attention, and automatization. The results identified three subgroups of dyslexic children: children with exclusive phonological deficits, children with combined deficits involving phonological, auditory, and magnocellular dysfunctions, and children with attention deficits. In the study by Menghini et al. (2010), 65 children and adolescents with DD and 60 typically developing peers were examined. The findings supported the presence of phonological deficits in all children with dyslexia, although only 18.3% of them exhibited exclusively this deficit. A total of 76.6% of the children with dyslexia showed multiple deficits, such as in executive functions, visuospatial perception, attention, memory, and motion detection, providing strong support for the notion that dyslexia is often accompanied by various cognitive deficits and is not limited to phonological difficulties. Furthermore, the study by White et al. (2006) examined the role of sensorimotor deficits in dyslexia, investigating the cerebellar, magnocellular, and phonological deficit hypotheses. The sample included 23 children with DD and 22 typically developing children, matched for age and non-verbal intelligence. The findings indicated that all children with dyslexia exhibited deficits in phonological tasks, and a small subgroup of children showed visual difficulties, particularly in visual stress tasks. However, some of these studies were conducted in opaque orthographic systems (White et al., 2006), others in transparent ones (Menghini et al., 2010) and others in intermediate ones (Heim et al., 2008) and this may have influenced their findings. Additionally, the sample of the aforementioned studies was rather small. Examining many children with dyslexia is considered a critical factor in distinguishing subtypes of the disorder as some cases of dyslexia may occur very rarely and may not be detected in small samples.

Therefore, it is important to conduct studies in different linguistic systems, which will fill the above gaps in literature. Given the uniqueness of the Greek language, which is classified as a transparent orthographic system, and the fact that no study in the Greek literature has simultaneously investigated a wide range of cognitive domains, such research is necessary. This study would aim to identify subtypes of dyslexia in the Greek language and explore the possibility of multiple cognitive subtypes of dyslexia in children, as supported by contemporary multiple deficit models (Pennington et al., 2012; van Bergen et al., 2014b). The aim of this study was to identify if Greek-native children with dyslexia can be distinguished into distinct categories based on their performance on various tasks that have been associated with the onset of dyslexia, indicating that dyslexics can be distinguished into distinct subtypes. A further aim of the study was to discuss current theoretical approaches and research findings that support the existence of multiple deficits in children with dyslexia.

Based on the preceding theoretical review, we formulated the general hypothesis of the study, according to which children with dyslexia are expected to present differentiated profiles based on which they can be distinguished into distinct subtypes. This general hypothesis leads to three predictions. According to the phonological deficit hypothesis (Bradley and Bryant, 1983; Snowling, 1995, 2000) and Frith’s (1999) causal framework for dyslexia, we expect that a distinct subtype of children with DD will be characterized based on their performance in the phonological domain (Prediction 1). Based on the other two general causal frameworks for the interpretation of dyslexia proposed by Frith (1999), namely the magnocellular deficit framework and the cerebellar deficit framework, we expect that a distinct subtype of children with DD will emerge based on their performance in processing speed and another subtype based on their performance in motor tasks (Prediction 2). In line with multiple deficit models (Pennington et al., 2012; van Bergen et al., 2014a), we expect that distinct subtypes of children with DD can be identified who will exhibit a combination of neurocognitive deficits (Prediction 3).

## 2 Materials and methods

### 2.1 Participants

The sample consisted of 101 children with dyslexia, 63 males and 38 females (age range 8–12 years, *M* = 11.15 years, SD = 0.88) who were attending the 3rd, 4th, 5th, and 6th grade of Greek primary school. Specifically, the sample consisted of six children aged 8–9 years (4 boys and 2 girls), four children aged 9–10 years (2 boys and 2 girls), 24 children aged 10–11 years (11 boys and 13 girls), and 67 children aged 11–12 years (46 boys and 21 girls). A convenience sampling approach (Creswell, 2012) was employed, as all students had to have been diagnosed with dyslexia by an official public diagnostic center for special educational needs. The selection of students was based on records from the Centers for Interdisciplinary Assessment, Counseling, and Support (KE.DA.SY.), following the acquisition of all necessary approvals from the appropriate authorities. The selection of students was based on records from the Centers for Interdisciplinary Assessment, Counseling, and Support (KE.DA.SY.), following the acquisition of all necessary approvals from the relevant authorities. According to Greek legislation, KE.DA.SY. conducts individual assessments of preschool and school-age students through interdisciplinary teams. The core composition of these teams includes a special education teacher, a psychologist, and a social worker. The special education teacher evaluates reading performance using the standardized Greek version of the Reading Test (Test-A) (Panteliadou and Antoniou, 2008), while the psychologist conducts cognitive assessments using the Greek adaptation of the Wechsler Intelligence Scale for Children (WISC-III) (Georgas et al., 1997). The participants in the present study received their diagnosis, according to the discrepancy criterion within a timeframe ranging from 1 to 3 years prior to the implementation of the study. Children who did not have Greek as their native language or lived in bilingual/multilingual family environments were not included in the sample. Additionally, comorbidity with other developmental or behavioral disorders served as a criterion for non-participation in the study.

### 2.2 Materials and procedure

Participants were given a series of tests assessing a wide range of abilities and skills which have been scientifically documented to be associated with the occurrence of DD. Specifically, the phonological, memory and attention abilities, the processing speed, motor, visual, and visual-motor skills were assessed. All tests were administered individually in a session that lasted 1.5–2 h. The same sequence of administration was followed for all children. All tests were administered and scored according to the instructions of their creators. Below are listed the tests administered by skill area.

Phonological abilities domain: to assess phonological awareness, the Greek version (Kassotaki-Maridaki, 1998) of the Non-word Reading test of “The Children’s Test of Non-word Repetition” (Gathercole et al., 1994) was administered. To evaluate verbal short-term memory (Morris, 1999), the “Forward digit span” subtest of the Greek version of the Wechsler Intelligence Scale for Children-Fifth Edition (WISC-V) (Stogiannidou, 2017) was administered.

Attention abilities domain: for the assessment of auditory attention, the “Auditory attention range” task from the psychometric Test of Detection and Investigation of Attention and Concentration for Primary School Students (Simos et al., 2007) was administered. For the assessment of visual-spatial attention, the subtest “Map Mission” from the Greek standardization (Malegiannaki et al., 2015) of the Test of Everyday Attention for Children (TEA-Ch) (Heaton et al., 2001; Malegiannaki et al., 2019; Manly et al., 2002) was administered. Immediate Recall was assessed using the Attention-Enhanced Composite of the Greek-standardized version of the “Detroit Test of Learning Aptitude (DTLA-4)” (Tzouriadou et al., 2008). This composite comprises the subtests: Design sequences, Sentence reproduction, Reversed letters, Design reproduction, Word sequences, and Story sequences.

Memory abilities domain: for the assessment of long-term memory, the subtest “Opposite meanings” from the DTLA-4 was administered (Tzouriadou et al., 2008) and for working memory, the subtest “Backward digit span” from the WISC-V (Stogiannidou, 2017) was administered. For the assessment of immediate verbal memory, the subtest “Word sequences” from the DTLA-4 (Tzouriadou et al., 2008) was administered. For the assessment of auditory memory, the subtest “Reversed letters” from the DTLA-4 was administered (Tzouriadou et al., 2008) and for assessment of visual long-term memory, the Rey-Osterrieth Complex Figure Test was administered using the recall reproduction task (ROCF-Recall) (Lezak, 1995; Osterrieth, 1944; Rey, 1941, 1959).

Motor skills domain: fine motor skills were assessed using the subtest “Design sequences” from the DTLA-4 (Tzouriadou et al., 2008). The “Balance duration” task from the array of paracephalic tests by Dow and Moruzzi (1958) and the “Balance on the dominant leg” task from the Bruininks-Oseretsky Test of Motor Proficiency (BOTMP) (Bruininks, 1978) were administered to assess static balance. For the assessment of dynamic balance, three tasks (Walking forward, Walking forward “heel-toe” in one line of walking, and Walking backward) from the Movement Assessment Battery for Children (M-ABC) (Henderson and Sugden, 1992) were administered.

Processing speed: processing speed was assessed using the Coding subtest from the Greek version of WISC-V (Stogiannidou, 2017).

Visual skills domain: for the assessment of visual processing, the subtest “Symbolic relations^1^” from DTLA-4 (Tzouriadou et al., 2008) was administered.

Visual-motor skills domain: for the assessment of visual-motor skills, the Rey-Osterrieth Complex Figure Test (ROCF-Copy) (Lezak, 1995; Osterrieth, 1944; Rey, 1941, 1959) and the “Design reproduction” from the DTLA-4 (Tzouriadou et al., 2008) were administered. Visual-motor coordination was assessed with the contrasting composite of the Motor-Enhanced of DTLA-4 (Tzouriadou et al., 2008). This specific composition consists of the subtests: Design sequences, Reversed letters, Design reproduction, and Story sequences.

The psychometric properties of all administered tests were examined through a series of reliability analyses. The calculated Cronbach alpha reliability coefficients ranged from 0.75 to 0.98 indicating high internal consistency in all tests.

Table 1 summarizes by domain the abilities and skills assessed through the 18 tests administered in this study.

**TABLE 1:** The tests administered in this study by skills domain.

Domain	Skills/abilities	Tests
Phonological	Phonological awareness	Reading Greek pseudowords
	Verbal short-term memory	Forward digit span
Attention	Auditory attention	Auditory attention range
	Visual spatial attention	Map mission
	Immediate recall	Enhanced attention
Memory	Long-term memory	Opposite meanings
	Working memory	Backward digit span
	Immediate verbal memory	Word sequences
	Auditory memory	Reversed letters
	Visual long-term memory	ROCF-Recall
Motor	Fine motor skills	Design sequences
	Static balance	Balance duration and balance on the dominant leg
	Dynamic balance	Walking forward, walking forward “heel-toe” in one line of walking, and walking backward
Processing speed	Processing speed	Coding
Visual	Visual processing	Symbolic relations
Visual-motor	Visual-motor skills	Design reproduction ROCF-Copy
	Visual-motor coordination	Motor

**FIGURE 1:**
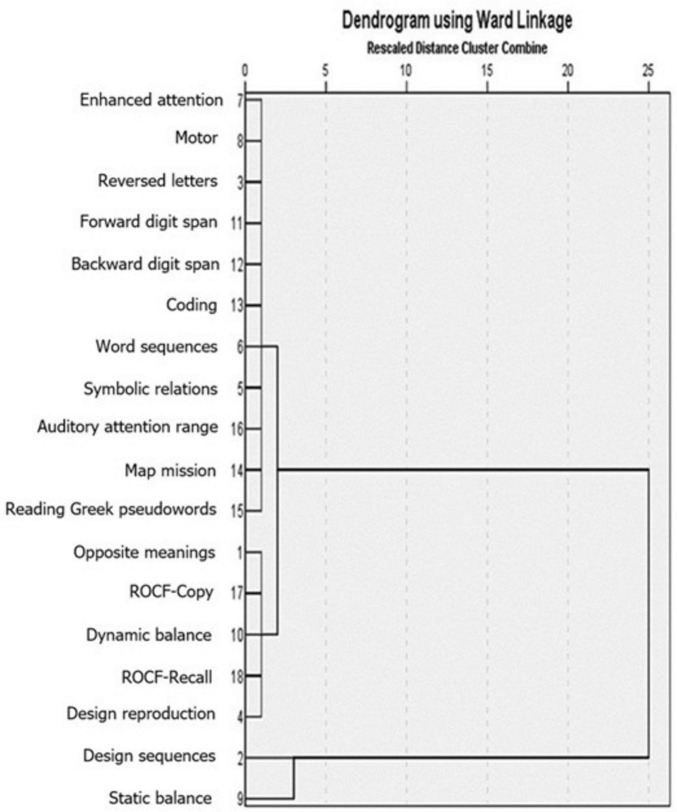
The dendrogram of the hierarchical cluster analysis by variable, in which the categorization of the 18 variables into 3 clusters is displayed.

All statistical analyses were conducted using SPSS 26. Descriptive statistics representing the participants’ performance in all 18 tests were calculated, followed by two Hierarchical Cluster Analyses. In these analyses agglomerative clustering was deemed the preferred method as it represents a sophisticated technique that starts with considering individual data points as independent clusters and proceeds with successive mergers thus combining the most similar pairs of clusters together (Murtagh and Legendre, 2014). Specifically, the first hierarchical cluster analysis by variables (i.e., the 18 tests administered) was performed with a view to identifying clusters representing distinct domains of skills. Since the number of clusters cannot be predetermined, we selected the largest ones identified in the dendrogram (Figure 1), which amounted to three. Following this, hierarchical cluster analysis by cases was conducted with a view to allocating the participating students to the three previously identified clusters. In these analyses, we utilized Ward’s method of calculating distance between clusters as it has been advocated as a promising method for establishing highly homogeneous groups (Ward, 1963 as cited in Murtagh and Legendre, 2014). Finally, a series of one-way analyses of variance (ANOVA) were used to compare the identified sets of participants in each of the 18 tests administered.

## 3 Results

### 3.1 Descriptive analysis

Table 2 shows the means, standard deviations, and the range of scores obtained in all tests administered.

**TABLE 2:** Means, standard deviations, minimum, and maximum scores per test.

Test	*M*	SD	Min.	Max.
Reading Greek pseudowords	13.49	5.22	2	26
Forward digit span	6.84	1.28	4	10
Auditory attention range	13.66	2.33	9	18
Map mission	11.20	4.10	2	25
Enhanced attention	3.07	2.43	1	9
Opposite meanings	24.81	7.50	7	46
Backward digit span	6.99	1.72	4	12
Word sequences	7.08	2.99	1	23
Reversed letters	2.60	0.90	1	5
ROCF-Recall	21.34	9.15	1.5	36
Design sequences	113.49	12.50	85	137
Static balance	134.51	64.24	44	395
Dynamic balance	18.86	4.40	9	27
Coding	8.16	2.36	3	15
Symbolic relations	14.14	5.45	4	27
Design reproduction	24.86	15.04	0	63
ROCF-Copy	29.49	6.15	5.5	36
Motor	3.71	2.63	1	9

### 3.2 Hierarchical cluster analysis by variables

Figure 1 represents a dendrogram depicting three distinct clusters. The first cluster includes most of the tests, specifically: Enhanced attention, Motor, Reversed letters, Forward digit span, Backward digit span, Coding, Word sequences, Symbolic relations, Auditory attention range, Map mission, and Reading Greek pseudowords. The second cluster incorporates five tests: Opposite meanings, ROCF-Copy, Dynamic balance, ROCF-Recall, and Design reproduction. The third and final cluster comprises two tests, Design sequences and Static balance.

Table 3 shows the three clusters generated. The first cluster includes six skill domains, namely memory, attention, processing speed, phonological domain, visual-motor, and visual domain. In the second cluster, three skills are included, namely memory, the motor, and the visual-motor domain. Finally, the third cluster includes only one skill domain, the motor. Specifically, the first and second clusters are distinguished by their performance in tasks assessing the domains of memory and visual motor. Additionally, the first cluster also stood out for its performance in the domains of attention, processing speed, phonological, and visual domain. The second cluster, apart from its performance in tasks assessing memory and visual-motor domain, was also distinguished by its performance in the motor domain.

**TABLE 3:** The three clusters by skills domain, that resulted from the hierarchical cluster analysis by variable.

	Cluster 1	Cluster 2	Cluster 3
Domain	Memory	Memory	Motor
	Attention	Motor	
	Processing speed	Visual – motor	
	Phonological		
	Visual motor		
	Visual		

### 3.3 Hierarchical cluster analysis by cases

**FIGURE 2:**
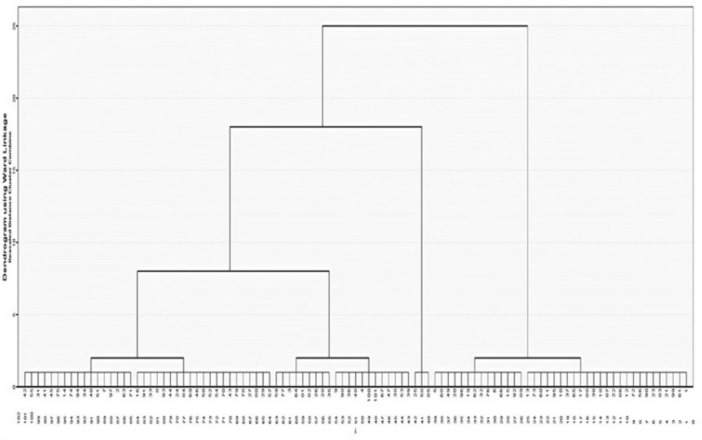
The dendrogram of the hierarchical cluster analysis by case, displaying the number of dyslexic children in each cluster.

Figure 2 presents the dendrogram that emerged which depicts three groups of participants.

Table 4 shows the number (*N*) and percentage (%) of children belonging to each of the three clusters that emerged. In the first cluster, which includes the domains of memory, attention, processing speed, phonological, visual-motor, and visual, there are 39 out of 101 children in the sample (38.61%). The second cluster, encompassing three skills domains (memory, motor, and visual-motor), consists of 59 children (58.42%). The third cluster, composed of 3 children (2.97%), includes only the motor domain.

**TABLE 4:** The number and percentage of participants per cluster.

	*N*	%
Cluster 1	39	38.61
Cluster 2	59	58.42
Cluster 3	3	2.97
Valid	101	100
Missing	0	0

### 3.4 Analysis of the characteristics of each cluster

**FIGURE 3:**
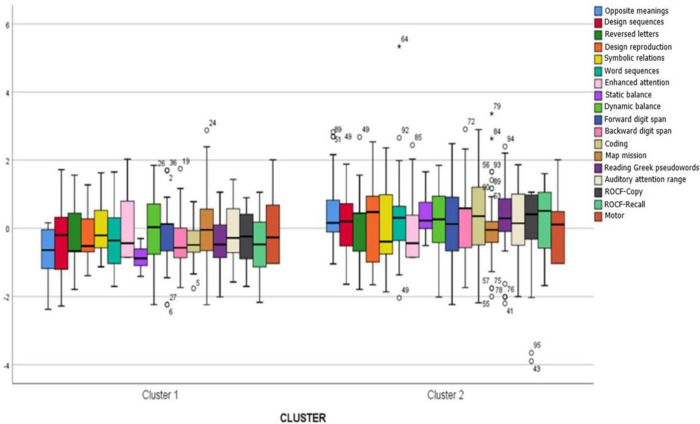
Comparison of the performances of children from the 1st and 2nd clusters in the administered tests.

In Figure 3, the performances of students from the first and second clusters are visualized in all administered tests. The performances of students from the third cluster were chosen not to be visualized due to the small number of students included in it (*N* = 3). These specific students are distinguished in a separate cluster (cluster 3) due to their differentiated performances in the motor domain, specifically in the Design sequences and Static balance tests (see Figure 2) compared to the rest of the students. Students belonging to the first cluster (*N* = 39) scored lower in all seven domains (phonological, processing speed, attention, memory, visual, motor, and visual-motor) compared to students belonging to the second cluster (*N* = 59), although the differences in performances between the two clusters were statistically significant in five of seven domains. Specifically, in the domains of memory, motor and visual-motor skills, processing speed, and phonological skills (see Table 5).

**TABLE 5:** Means and standard deviations of the scores of the dyslexics on the 18 tests examined by cluster.

Tests	Cluster 1 (*N* = 39)		Cluster 2 (*N* = 59)		ANOVA
	***M***	**SD**	***M***	**SD**	***F***
Opposite meanings	19.90	5.29	28.36	6.92	22.77*
Design sequences	108.79	13.40	116.05	11.16	5.51*
Reversed letters	2.41	0.82	2.81	0.86	8.44*
Design reproduction	20.00	10.25	27.71	17.15	3.62*
Symbolic relations	13.82	4.06	14.46	6.31	0.39
Word sequences	6.28	2.46	7.68	3.24	3.04
Enhanced attention	3.10	2.51	3.14	2.42	0.79
Static balance	79.62	20.40	158.58	35.00	182.17*
Dynamic balance	18.67	4.54	19.36	4.19	4.75*
Forword digit span	6.62	1.29	7.02	1.27	1.43
Backword digit span	6.44	1.50	7.41	1.79	4.54*
Coding	7.15	1.41	8.81	2.68	6.44*
Map mission	11.13	4.73	11.22	3.78	0.03
Reading Greek pseudowords	11.08	4.38	15.19	5.22	8.72*
Auditory attention	13.36	2.15	13.98	2.43	2.46
ROCF-Copy	27.76	5.09	30.40	6.67	3.12*
ROCF-Recall	16.96	8.13	23.78	8.82	9.36*
Motor	3.72	2.61	3.71	2.73	0.00

*p < 0.05.

As can be seen on Table 5, the analysis of variance revealed that the performances of students in the first cluster are characterized by statistically significant differences compared to the performances of students in the second cluster. Specifically, students in the first cluster did not show statistically significantly lower performances in two out of seven skill domains, namely in the attention domain and visual domain.

More specifically, students in the first cluster recorded statistically significantly lower performances in long-term memory (task “Opposite meanings,” *F*(2,98) = 22.77, *p* < 0.05), fine motor skills (task “Design sequences,” *F*(2,98) = 5.51, *p* < 0.05), auditory memory (task “Reversed letters,” *F*(2,98) = 8.44, *p* < 0.05), visual-motor skills (tasks “Design reproduction,” *F*(2,98) = 3.62, *p* < 0.05, and “ROCF-Copy” *F*(2,98) = 3.12, *p* < 0.05), static balance (task “Static balance,” *F*(2,98) = 182.17, *p* < 0.05), dynamic balance (task “Dynamic balance,” *F*(2,98) = 4.75, *p* < 0.05), working memory (task “Backward digit span,” *F*(2,98) = 4.54, *p* < 0.05), processing speed (task “Coding,” *F*(2,98) = 6.44, *p* < 0.05), phonological awareness (task “Reading Greek pseudowords,” *F*(2,98) = 8.72, *p* < 0.05), and visual long-term memory (task “ROCF-Recall,” *F*(2,98) = 9.36, *p* < 0.05).

Table 6 presents the means, standard deviations, and range of participants’ age per cluster and overall. ANOVA revealed significant differences between the ages in the three clusters. More specifically, it revealed that the mean age of participants in the first cluster differs statistically significantly from the mean ages of participants in the other two clusters, indicating that majority of younger participants were classified into the first cluster.

**TABLE 6:** Means, standard deviations, and range of students’ age per cluster and overall.

	Age			
	***M***	**SD**	**Min.**	**Max.**
Cluster 1	10.85*	1.10	8.5	11.9
Cluster 2	11.31	0.66	9.3	11.9
Cluster 3	11.37	0.06	9.5	11.9
Total	11.15	0.88	8.5	11.9

*p < 0.05.

## 4 Discussion

According to the general hypothesis of the study, we expected that children with dyslexia would present differentiated profiles based on which they could be distinguished into distinct subtypes. Based on our first prediction we expected that a distinct subtype of children with DD would be characterized based on their performance in the phonological skills area. The results of this study did not confirm this hypothesis. Children belonging to the first cluster, in addition to differential performance in tests assessing the phonological skills domain, showed differential performance in tests assessing memory, attention, processing speed, visual and visual-motor skills domain.

Our results, regarding the presence of additional deficiencies concurrent with variations in dyslexic students’ performance in the phonological skills domain, are in line with recent research findings (Constantinidou and Stainthorp, 2009; Douklias et al., 2009; McGrath et al., 2011; Lewandowska et al., 2014; O’Brien and Yeatman, 2021; Ruffino et al., 2010; Schuchardt et al., 2008). The results indicate the simultaneous existence of other deficits beyond the phonological in children with dyslexia.

However, our results are not in line with the findings of other studies (Perez et al., 2012; Saksida et al., 2016; Soriano-Ferrer et al., 2014), which present phonological deficits as the sole impairment in children with dyslexia. A distinct group of dyslexics with deficits only in the phonological skills domain was also identified in the studies of Borleffs et al. (2018), Menghini et al. (2010), Ramus et al. (2003) and White et al. (2006).

The differences in our results compared to those of other studies could be attributed to various reasons. A possible reason is the transparency of the spelling system between languages, a factor that affects phonological processing. Phonological deficits appear to be more frequent and pronounced in opaque orthographic systems, such as the English language (Martin et al., 2016). In the studies conducted by Ramus et al. (2003) and White et al. (2006), the participants were native English speakers. The English orthographic system is considered one of the opaquest orthographic systems. However, the Greek orthographic system is the second most transparent orthographic system among European languages (Seymour et al., 2003) and studies have shown that in such systems, the rate of phonological deficits is significantly lower as decoding is easier (Ziegler and Goswami, 2005).

An additional factor that may affect the differences between the studies are sample sizes. In the studies of Ramus et al. (2003) and White et al. (2006), the sample size was relatively small, especially considering the administration of numerous diverse tasks. However, in our research we examined a very large sample of dyslexics, which provides higher power to the analysis. Another factor that differentiates the findings could be the variation in the chronological age of the samples. For example, the study by Ramus et al. (2003) focused on university students, while the research by Menghini et al. (2010) included both children and adolescents. The older age of the participants may be associated with the acquisition of more advanced skills, potentially influencing the manifestation of phonological deficits compared to younger children. Recent studies have shown that education affects the brain’s language network, as learning to read supports the development of more advanced phonemic skills through experience (Łuniewska et al., 2019). Therefore, based on all the above findings we could assume that phonological deficits appear to be less pronounced in older children compared to younger ones.

Furthermore, the differentiation of our results from the findings of Borleffs et al. (2018) could be attributed to the sample characteristics in their study. The children participating in their research were at high risk of dyslexia but had not received a formal diagnosis. In contrast, in our study, all children had a confirmed diagnosis of dyslexia.

Additionally, the absence in our study of a distinct cluster of children characterized only by their performance in phonological skills could also be explained from the perspective that reading is a factor influencing an individual’s phonological abilities (Menghini et al., 2010). The improvement of reading with age and progress in school enhances children’s phonological performance. This may explain why phonological deficits are less apparent in older age groups. According to research (Papadimitriou and Vlachos, 2014), phonological awareness is a strong predictor of reading ability in first grade, but its predictive power decreases in second grade as other factors come into play. Our findings seem to align with this perspective. Most of the younger children in our study belong to the cluster characterized, among other factors, by its performance in the phonological domain (cluster 1). In contrast, the other two clusters, which include the older children in the study, did not demonstrate notable performance in phonological skills.

Another factor that might have contributed to the absence of a distinct group of children characterized solely by their performance in phonological skills is the lack of sufficient tasks in our study that assess this specific domain. Based on the extensive literature on the phonological deficit in dyslexia, we expected that such a distinct subtype would be easily distinguishable without the administration of several relevant tests. For this reason, we did not include more tasks assessing this specific domain but chose to focus on tasks investigating other cognitive domains, for which the research evidence is more limited.

The second prediction of this study predicted that a distinct subtype of children with DD would be identified, differing based on their performance in processing speed, and another subtype based on their performance in motor tasks. Our findings partially confirm our second research hypothesis in relation to both aspects.

Regarding the first aspect of the prediction, our results showed that such differentiation is observed in 38.61% of the children comprising the first cluster. This percentage constitutes a distinct subtype of dyslexics that exhibits differences in processing speed simultaneously with variations in memory, visual, visual-motor, attentional, and phonological skills area. Our results are consistent with the findings of recent studies (Moll et al., 2016; Niolaki et al., 2014; O’Brien et al., 2012; O’Brien and Yeatman, 2021) that identified that dyslexics often present additional deficits beyond the processing speed deficit.

As for the second aspect of the second prediction, participants in the third cluster showed differences in their performance in motor tasks. Although this cluster constitutes only 2.97% of the sample, all statistical analyses indicated that it represents a distinct subtype among children with dyslexia, differing in static balance and fine motor skills. Our findings are in line with the findings of previous studies (Getchell et al., 2007; Iversen et al., 2005; Kirby et al., 2008; Petropoulou, 2011) which showed difficulties in motor tasks for children with dyslexia. However, our findings differ partially from those of other studies (Needle et al., 2006; Ramus et al., 2003), possibly due to sample differences. In the study by Needle et al. (2006), the sample consisted of adults with dyslexia, and in the research by Ramus et al. (2003), most participants exhibited comorbidity with another disorder. However, in our study, the sample consisted exclusively of children, and comorbidity with another disorder served as a reason for exclusion from the sample.

The third prediction of the study predicted that distinct subtypes of children with DD would be identified, showing a combination of neurocognitive deficits. This hypothesis was fully confirmed as two large and distinct clusters of dyslexic children with a combination of deficits were identified. In the first cluster, which constitutes 38.61% of the sample, children exhibited difficulties in the domains of memory, attention, processing speed, phonological skills, visual processing, and visual-motor skills. In the second cluster (58.42%), children had difficulties in the skills domains of memory, motor, and visual-motor.

Our findings are in line with the results of recent studies (Heim et al., 2008; Lewandowska et al., 2014; Menghini et al., 2010; Soriano-Ferrer et al., 2014; O’Brien and Yeatman, 2021) indicating the simultaneous presence of various deficits in several domains (attention, memory, visual, motor, etc.) in dyslexic children. Such findings support the multiple deficits model for DD (Pennington et al., 2012; van Bergen et al., 2014b).

In conclusion, our findings indicate that children with dyslexia can be differentiated into subtypes based on their performance in cognitive tasks. These subtypes are not characterized by a single cognitive deficit but by deficits in various domains, which should be systematically assessed during the diagnostic process. According to neuroscientific research, the diverse clinical manifestations of the disorder can be interpreted by the different case-specific anatomical and/or functional organization of brain networks and the consequences of these neurobiological variations on language and/or other cognitive functions. Additionally, the comorbidity of developmental disorders is explained by multiple deficit models (van Bergen et al., 2014a) through the existence of certain “common risk factors” (Papanikolaou et al., 2017). According to these models, there are genetic and cognitive risk factors as well as protective factors against risks. Some factors are distinct for each of the disorders, while others are shared. Therefore, DD represents a complex and heterogeneous disorder, requiring a systematic investigation of the specific characteristics of each child.

Although the results of this study provided significant information about the neurocognitive deficits exhibited by students with DD, we consider that the present study is subject to few limitations. According to the initial research hypothesis, we expected to identify an unambiguous and distinct subtype of children with dyslexia characterized by their performance in the phonological skills area. This was the reason we did not administer more tests to evaluate this specific area. Instead, this study focused on the co-evaluation of other neurocognitive domains for which there is not as strong a research foundation. This may have influenced the research results and led to the absence of a distinct cluster of students with dyslexia characterized by difficulties in the phonological domain.

An additional limitation is the lack of assessment of the reading ability of children with dyslexia and, consequently, the identification of the specific reading characteristics of participants in each cluster. Due to the large number of tests administered to assess various skills domains, there was not enough time to administer additional tests that would provide information about the reading performance of students in each cluster and the possible reading domains (accuracy, fluency, and comprehension) that are deficient.

The results of recent studies (Heim et al., 2008; Menghini et al., 2010; van Bergen et al., 2014a), as well as the findings from our research, provide evidence for the complex nature of DD. Children with DD, in addition to phonological deficits simultaneously exhibit deficits in other skills domains, such as attention, memory, visual, motor skills, etc. Therefore, there is a pressing need to enhance the diagnostic process by administering tests that assess a broader range of abilities. Administering tests that evaluate not only intelligence and reading abilities, but additional cognitive and motor skills would assist in describing a comprehensive individual neurocognitive profile.

Moreover, when describing the cognitive profile, it is important to consider the influence of the transparency of the orthographic system to which the child with dyslexia belongs, as variations are observed across different orthographic systems (Diamanti, 2010). In transparent systems, such as Greek, where there is a consistent correspondence between letters and phonemes, decoding becomes relatively easier, even for people with dyslexia. However, this may imply that these people show more pronounced difficulties in other domains, such as processing speed, attention, etc. On the contrary, in most opaque systems, such as English, the grapheme-phoneme correspondence is more complex. As a result, students with dyslexia face increased difficulties in reading and spelling.

Additionally, the finding of distinct subtypes in DD highlights the need to design and implement individualized educational interventions that respond to the strengths and weaknesses of each student. Until recently, most interventions focused on the phonological domain. However, the findings of the present study, combined with evidence from other studies (Heim et al., 2008; Menghini et al., 2010; White et al., 2006), indicate that individuals with dyslexia exhibit multiple deficits. Identifying and understanding the individual characteristics of students with dyslexia can contribute to the development of effective and targeted educational intervention programs (Pacheco et al., 2014) and allow the implementation of targeted cognitive and metacognitive strategies, improving reading comprehension and overall school performance of students (Moutsinas et al., 2019). The primary aim of these differentiated interventions based on the identified subtypes must be to prevent the consolidation of difficulties related to dyslexia.

Overall, our findings support the results of recent studies suggesting that DD constitutes a complex and heterogeneous disorder (Heim et al., 2008; Jednoróg et al., 2014; Menghini et al., 2010; O’Brien and Yeatman, 2021; Pennington, 2006; van Bergen et al., 2014a). This disorder is characterized by a multifactorial deficit rather than isolated impairments (Stanovich, 1988; Tallal, 1980; Nicolson and Fawcett, 1990; Nicolson et al., 2001). The majority of individuals with DD seem to exhibit a combination of neurocognitive deficits, with some cases also involving individuals displaying isolated deficiencies. Concurrently, these findings are consistent with recent neurobiological evidence suggesting that various cognitive subtypes of the disorder show differences in the structure and, consequently, the functioning of the brains of individuals with dyslexia. This supports contemporary models of multiple deficits in explaining the etiology of DD.

## Data Availability

The raw data supporting the conclusions of this article will be made available by the authors, without undue reservation.

## Footnotes

1 According to the developers of the DTLA-4, the Symbolic Relations subtest assesses non-verbal reasoning. In the present study, it was used to evaluate visual processing, as the ability for non-verbal logic appears to be derived from visual processing (Landy and Goldstone, 2007).
