# Supplementary material for: Are there distinct subtypes of developmental dyslexia?
Source: Front Behav Neurosci. 2025 Jan 3;18:1512892. doi: 10.3389/fnbeh.2024.1512892 (PMC11739093; doi:10.3389/fnbeh.2024.1512892)
Supplement: Supplementary file 1 [file Data_Sheet_1.pdf]

Supplementary Material

Administered Tests: Procedure and Scoring

Below is the procedure and scoring method for all the tests administered to the participants of our study.

PHONOLOGICAL DOMAIN ASSESSMENT TESTS

Phonological Awareness: *Reading Greek Nonwords*

The test is based on the Greek version (Kassotaki-Maridaki, 1998) of the Nonword Reading test from "The Children's Test of Nonword Repetition" (Gathercole et al., 1994).

Procedure: The test consists of 40 nonwords, divided into four groups of 10 based on syllable count: two, three, four, and five syllables. After informing the child that the words presented are not "real," they are instructed to read each word as quickly and accurately as possible within 45 seconds, starting at the top of the list and proceeding downward.

Scoring: Each correct answer is scored one and an incorrect answer is scored zero. The maximum score for this test is 40 points.

Short-term phonological memory: *Forward digit span*

This subtest is derived from the Greek version of the Wechsler Intelligence Scale for Children-Fifth Edition (WISC-V) (Stogiannidou, 2017).

Procedure: The test has eight questions with two sets of numbers each with the same number of digits. The first set is considered attempt 1 and the second set is considered attempt 2. After reading the sets of digits to the child (1 at a time), at a rate of one digit per second, we ask them to repeat it from beginning to end. The sets increase by one digit at a time. Even if the child succeeds on attempt 1, we grant both attempts.

Scoring: Each question is scored two points if the child succeeds in both attempts, one point if the child succeeds on the first attempt and zero if the child fails on both attempts of the question. After failure in both attempts of the same question, the procedure is discontinued. The maximum possible score is 16 points.

ATTENTION DOMAIN ASSESSMENT TESTS

Auditory attention: *Auditory attention range*

This test is a subscale from the psychometric Test of Detection and Investigation of Attention and Concentration for Primary School Students (Simos et al., 2007).

Procedure: The examiner reads in normal speech, one sentence at a time, and asks the child to repeat the sentence word for word. The sentences are drawn from a list of nine sentences of increasing length, and repetition of a sentence by the examiner is not allowed. If a child in grade 4 and above does not accurately repeat the third or fourth sentence (baseline rule), the examiner administers the first and second sentences before proceeding to the fifth sentence.

Scoring: For each accurate repetition of a sentence, the examiner awards two points. If the child makes one or two errors, the examiner gives one point and zero if the child makes more than two mistakes. Changing, adding or omitting a word or transposing two words counts as an error. An error is defined as a substitution, addition, or omission of a word, or the transposition of two words. The test is discontinued if the child provides two consecutive zero-score responses. The maximum possible score for this test is 18 points.

Visual spatial attention: *Map Mission*

The Map Mission is a subtest from the Greek standardization (Malegiannaki et al., 2015) of the Test of Everyday Attention for Children (TEA-Ch) (Heaton et al., 2001; Malegiannaki et al., 2019; Manly et al., 2002).

Procedure: The child had in front of him a laminated map of the city of Thessaloniki (Greece) in A3 size (29.7 x 42 cm) on which he had to circle as many symbols indicating a restaurant (knife-fork) within the time limit of 60 seconds. The symbols are relatively small and scattered among other distracting symbols.

Scoring: For each correct symbol the child found, the examiner gives one point. The maximum number of points is 60.

Immediate recall: *Enhanced attention*

Enhanced Attention is a Composite from the Greek standardized version of the "Detroit Test of Learning Aptitude (DTLA-4)" (Tzouriadou et al., 2008). This composite comprises six subtests: Design sequences, Sentence reproduction, Reversed letters, Design reproduction, Word sequences, and Story sequences.

1) Design sequences

Procedure: The test consists of 12 pictures, each containing a series of abstract drawings/symbols and six cubes with one of the abstract symbols drawn on each seat. After showing the child a picture for five seconds, the examiner removes the picture and asks the child to place the cubes in such a way that the picture he saw with the exact order of the symbols is formed. In the example, the examiner lets the child see the picture throughout the entire attempt. If the child finds the correct pattern and places the cube correctly then the examiner continues to the second cube, if not he/she asks the child to try again. Once the child has placed both cubes correctly, the examiner moves on to the main test and asks the child to make the symbol strings without looking at the picture book. The child is allowed to look at the picture book for each symbol row up to three times.

Scoring: Each correct design is awarded one point regardless of first, second or third attempt. If the child fails to place the drawings correctly after the third attempt, the examiner scores one point for correct drawings and zero points for incorrect ones. The maximum total number of points is 147.

2) Sentence reproduction

Procedure: The subtest consists of 35 sentences which the child has to repeat after the examiner. If the child does not answer correctly, the examiner repeats the sentence. If the child reproduces the sentence correctly, the examiner moves on to the next sentences. For children aged 8.0-9.11 the starting point for the test is question 1, and for children aged 10.0-14.11 the starting point is question 10.

Scoring: Each sentence that the child reproduces correctly is awarded one point. If he/she makes a mistake, i.e. omits a word, changes the order of the words or replaces a word with another, he/she is

given a zero. The administration stops if the child gives five consecutive incorrect answers. The maximum possible score for this test is 35 points.

3) Reversed letters

Procedure: The examiner reads clearly and at a rate of one letter per second a series of letters which the child must write backwards. Each letter must be written in a small box, without touching or coming out of it.

Scoring: If the child has written the letters correctly and in reverse order, the answer gets one point otherwise it gets zero. A zero is also given to the answer that was spelled correctly but from right to left. The maximum number of points is 85.

4) Design reproduction

Procedure: The examiner shows the child an image of a geometric shape, then removes it, asking the child to draw it from memory without using an eraser. After the example, the test is administered.

Scoring: The test is scored based on standard blueprints included in the examiner's guide. The points that can be awarded to each drawing are zero if the drawing is too far off or the child fails to represent it at all, one and two points for drawings that look like the one seen, and three points if the child draws it identical to the template. The administration stops if the child gets three consecutive drawings wrong. The maximum possible score is 69 points.

5) Word sequences

Procedure: The test consists of 30-word sequences that increase in length progressively. The child is asked to repeat the sequences in the exact order they are read aloud. For children aged 8.0-9.11 the starting point is question 1 (1st row), while for children aged 10.0-14.11 the starting point is question 12 (12th row). The maximum number of attempts is five.

Scoring: Each correct answer is awarded one point. If the child omits a word, says a synonym or says the words in the wrong order, a zero is awarded. The maximum possible score for this test is 30 points.

6) Story sequences

Procedure: The subtest consists of eight rows of cards that if the child puts them in order, a story is formed for each row. The number of cards gradually increased in each row.

Scoring: Each picture that the child places correctly is scored with one point and zero for the pictures that he/she did not place in the correct order. The maximum possible score for this test is 48 points.

MEMORY DOMAIN ASSESSMENT TESTS

Long-term memory: *Opposite meanings*

This test is a subtest of the DTLA-4 (Tzouriadou et al., 2008).

Procedure: It consists of 55 words for which the child is asked to find their opposites. For children aged 8.0-9.11 the starting point is question 1 (1st row), while for children aged 10.0-14.11 the starting point is question 12 (12th row). If the child cannot understand the test and answers incorrectly then only the first five questions are administered.

Scoring: Each correct answer is awarded one point. If the child omits a word, says a synonym or says the words in the wrong order, the child is awarded zero points. The maximum possible score for this test is 55 points.

Working memory: *Backward digit span*

This subtest is derived from the WISC-V (Stogiannidou, 2017).

Procedure: In this test there are seven pairs of rows. The first row is considered as attempt 1 and the second row as attempt 2. The child is read a series of numerical digits at the rate of one digit per second and asked to repeat it from the end to the beginning. The rows of digits are increased by one digit at a time.

Scoring: Each question scores two points if the child succeeds in both attempts, one point if the child succeeds on the first attempt and zero if the child fails on both attempts of the question. After failure in both attempts of the same question, the procedure is discontinued. The maximum possible score for this test is 14 points.

Immediate verbal memory: *Word sequences*

This test is a subtest of the DTLA-4 (Tzouriadou et al., 2008). For details on the procedure and scoring, please refer to page 4 above.

Auditory memory: *Reversed letters*

This test is a subtest of the DTLA-4 (Tzouriadou et al., 2008). For details on the procedure and scoring, please refer to page 4 above.

Visual long-term memory: *Recall reproduction of the Rey-Osterrieth Complex Figure (ROCF-Recall)*

In this test, the child was administered the Rey-Osterrieth Complex Figure (Lezak, 1995; Osterrieth, 1944; Rey, 1941, 1959).

Procedure: The examiner shows the child the ROCF, asking him to observe it very carefully and then draw it from memory, after removing it. The basic rectangular shape was 8.0 cm x 5.5 cm and was given to the children on a piece of paper measuring 30 cm x 21cm.

Scoring: The 18 structural elements of the scheme were evaluated for scoring. Each element drawn by the child is scored two points when it is correct and in the correct position. One point is awarded when it is distorted or when it is correct but in the wrong position. Half a point is awarded when it is distorted and in the wrong position. If the child omits an item or it is indistinguishable, it is scored as zero. The minimum scores the child can score is zero and the maximum is 36.

MOTOR DOMAIN ASSESSMENT TESTS

Fine motor skills: *Design sequences*

This test is a subtest of the DTLA-4 (Tzouriadou et al., 2008). For details on the procedure and scoring, please refer to page 3 above.

Static Balance: *Balance duration & Balance on the dominant leg*

For the assessment of Static Balance, two tasks were administered. Specifically, the "Balance duration" task from the array of paracephalic tests by Dow and Moruzzi (1958) and the "Balance on the dominant leg" task from the Bruininks-Oseretsky Test of Motor Proficiency (BOTMP) (Bruininks, 1978). For scoring, the total duration in seconds of the two tasks was recorded as the final score.

1) Balance duration

Procedure: The child is asked to stand upright for as long as he/she can with the legs together and the arms stretched out in front of him/her at shoulder height. An eye mask is placed over the eyes to keep them closed.

Scoring: The examiner records the time in seconds that the child managed to remain still in the requested position. If the child complains about the lack of vision due to the eye mask, the timer is stopped.

2) Balance on the dominant leg

Procedure: Initially, the examiner asks the child to stand upright on one leg, the dominant one, with their arms in the normal position. The other leg must be bent in such a way that the bent portion is parallel to the floor. Then, the examiner instructs the child to maintain this position.

Scoring: The examiner records the time in seconds that the child remained in this position, with a maximum time limit of ten seconds.

Dynamic balance: *Walking forward, Walking forward "heel-toe" in a straight line & Walking backward*

For the assessment of Dynamic balance, three tasks were administered: Walking forward, Walking forward "heel-toe" in a straight line, and Walking backward, from the Movement Assessment Battery for Children (MABC) (Henderson & Sugden, 1992). For scoring, the total number of steps across the three tasks was recorded.

1) Walking forward

Procedure: The child is asked to walk forward, at a normal pace, on a walking line five centimeters thick and 4.5 meters long, with his/her arms raised to shoulder height and each on his/her side.

Scoring: The examiner records the number of correct repetitive steps the child has managed to perform. The maximum number of steps is six.

2) Walking forward "heel-toe" in a straight line

Procedure: The examiner asks the child to take small steps forward, the size of a paw. When performing the steps, the toes of one foot must touch the heel of the other foot, i.e. heel-to-toe steps. Also, the child's hands should be raised to shoulder height and each on its side (right and left). The steps are performed on a walking line 4.5 meters long and five centimeters thick.

Scoring: The examiner records the number of correct repetitive steps performed by the child, with a maximum of six steps.

3) Walking backward

Procedure: The examiner asks the child to take small steps backwards, the size of a paw, i.e. the toes of one foot must touch the heel of the other foot (heel-to-toe steps). The steps are performed on a 4,5 m wide and 5 cm thick walking line.

Scoring: To score the test, the number of correct repeated steps backwards is calculated. The maximum number of backward steps is 15 steps.

PROCESSING SPEED DOMAIN ASSESSMENT TESTS

Processing speed: *Coding*

This subtest is derived from the WISC-V (Stogiannidou, 2017).

Procedure: The child is asked to copy symbols corresponding to simple geometric shapes using a key, within a time limit of 120 seconds. When the 120-second time limit expires, the examiner instructs the child to stop.

Scoring: One point is awarded for each symbol that is correctly drawn, without a rotation of ≤ 90º and within the time limit. Symbols that are incorrectly drawn, have a rotation of ≥ 90º, or are completed outside the 120-second time limit are scored as zero. The maximum total score is 117 points.

VISUAL DOMAIN ASSESSMENT TESTS

Visual processing: *Symbolic relations*

This test is a subtest of the DTLA-4 (Tzouriadou et al., 2008).

Procedure: The test consists of pages where each page presents a series of geometric shapes or linear designs with a missing part. The child must choose, from six possible designs, the one that completes the image.

Scoring: For each correct answer, the examiner awards one point, while for each incorrect answer, zero points are given. The test is discontinued if the child provides five consecutive incorrect answers. The maximum total score is 30 points.

VISUAL DOMAIN ASSESSMENT TESTS

Visual-motor skills: *ROCF-Copy & Design Reproduction*

Visual-motor skills were assessed using the ROCF Copying Test (ROCF-Copy) and the "Design Reproduction" subtest from the DTLA-4 (Tzouriadou et al., 2008).

1) ROCF-Copy

In this test, the child was administered the ROCF (Lezak, 1995; Osterrieth, 1944; Rey, 1941, 1959).

Procedure: The examiner shows the child the ROCF, asking him to observe it very carefully and then copy it. The basic rectangular shape was 8.0 cm x 5.5 cm and was given to the children on a piece of paper measuring 30 cm x 21 cm.

Scoring: The 18 structural elements of the scheme were evaluated for scoring. Each element drawn by the child is scored two points when it is correct and in the correct position. One point is awarded when it is distorted or when it is correct but in the wrong position. Half a point is awarded when it is distorted and in the wrong position. If the child omits an item or it is indistinguishable, it is scored as zero. The minimum scores the child can score is zero0 and the maximum is 36.

2) Design reproduction

This test is a subtest of the DTLA-4 (Tzouriadou et al., 2008). For details on the procedure and scoring, please refer to page 4 above.

Visual-motor coordination: *Motor*

The Motor-Enhanced Contrasting Composite of the DTLA-4 (Tzouriadou et al., 2008) consists of five subtests: Design sequences, Reversed letters, Design reproduction, and Story sequences. For details on

the procedure and scoring of the subtest "Design sequences", please refer to page 3, for "Reversed letters", "Design reproduction", and "Story sequences", please refer to page 4.

References

Bruininks, R. (1978). *Manual of Bruininks-Oseretsky Test of Motor Proficiency*. Circle Pines, MN: American Guidance Service.

Dow, R.S, and Moruzzi, G. (1958). *The Physiology and Pathology of the Cerebellum.* Minnesota Press

Gathercole, S.E., Willis, C.S., Baddeley, A.D. and Emslie, H. (1994). 'The children's test of nonword repetition: A test of phonological working memory', *Memory*, 2(2), pp. 103-127. doi: 10.1080/09658219408258940.

Heaton, S.C., Reader, S.K., Preston, A.S., Fennell, E.B., Puyana, O.E., Gill, N. and Johnson, J.H. (2001). 'The Test of Everyday Attention for Children (TEA-Ch): Patterns of Performance in Children with ADHD and Clinical Controls', *Child Neuropsychology*, 7(4), pp. 251-264. doi: 10.1076/chin.7.4.251.8736.

Henderson, S., & Sugden, D. (1992). *The Movement Assessment Battery for Children*. London: The Psychological Corporation.

Kassotaki - Maridaki, A. (1998). 'Short term memory of phonological information and reading achievement: An attempt to investigate their relationship', *Psychologia, 5*, 44–52. (In Greek).

Lezak, M.D. (1995). *Neuropsychological assessment*. 3rd edn. New York: Oxford University Press.

Malegiannaki, A.C., Aretouli, E., Metallidou, P., Messinis, L., Zafeiriou, D. and Kosmidis, M.H. (2019). 'Test of Everyday Attention for Children (TEA-Ch): Greek Normative Data and Discriminative Validity for Children with Combined Type of Attention Deficit-Hyperactivity Disorder', *Developmental Neuropsychology*, 44(2), pp. 189-202. doi: 10.1080/87565641.2019.1578781.

Malegiannaki, A.C., Metallidou, P. and Kiosseoglou, G. (2015). 'Psychometric properties of the Test of Everyday Attention for Children in Greek-speaking school children', *European Journal of Developmental Psychology*, 12(2), pp. 234-242. doi: 10.1080/17405629.2014.973842.

Manly, T., Anderson, V., Nimmo-Smith, I., Turner, A., Watson, P. and Robertson, I. (2002). The Differential Assessment of Children's Attention: The Test of Everyday Attention for Children (TEA-Ch), Normative Sample and ADHD Performance. *The Journal of Child Psychology and Psychiatry and Allied Disciplines*, 42(8), pp. 1065-1081. doi: 10.1017/S0021963001007909.

Osterrieth, P.A. (1944). Le test de copie d'une figure complexe. *Archives de Psychologie*, 30, pp. 286-356.

Rey, A. (1941). L'examen psychologique dans les cas d'encéphalopathie traumatique. (Les problems.). *Archives de Psychologie*, 28, pp. 215-285.

Rey, A. (1959). *Manuel: test de copie d'une figure complexe*. Paris: Centre de Psychologie Apliquee.

Simos, P., Mouzaki, A. and Sideridis, G. (2007). *Test of Detection and Investigation of Attention and Concentration for Elementary School Students*. Athens: Greek Ministry of Education. (In Greek).

Stogiannidou, A. (2017). *WISC-V GR (Wechsler Intelligence Scale for Children - 5th Edition)*. Athens: Motivo Ekdotiki. (In Greek).

Tzouriadou, M., Anagnostopoulou, E., Toutountzi, E. and Psoinou, M. (2008). *Detroit Test of Learning Aptitude (DTLA, DTLA-P: 3, DTLA-4)*. Thessaloniki, Greece: Aristotle University of Thessaloniki, Ministry of Greek Education. (In Greek).
